# In Vitro Characterization of Antioxidant, Antibacterial and Antimutagenic Activities of the Green Microalga *Ettlia pseudoalveolaris*

**DOI:** 10.3390/antiox12061308

**Published:** 2023-06-20

**Authors:** Andrea Vornoli, Teresa Grande, Valter Lubrano, Francesco Vizzarri, Chiara Gorelli, Andrea Raffaelli, Clara Maria Della Croce, Santiago Zarate Baca, Carla Sandoval, Vincenzo Longo, Luisa Pozzo, Cristina Echeverria

**Affiliations:** 1 Institute of Agricultural Biology and Biotechnology (IBBA), National Research Council (CNR), Via Moruzzi 1, 56124 Pisa, Italy; andrea.vornoli@ibba.cnr.it (A.V.); teresa.grande@unifi.it (T.G.); chiaragorelli97@gmail.com (C.G.); andrea1.raffaelli@santannapisa.it (A.R.); vincenzo.longo@ibba.cnr.it (V.L.); 2 Department of Experimental and Clinical Biomedical Sciences “Mario Serio”, University of Florence, Viale Morgagni 50, 50134 Florence, Italy; 3 Fondazione G. Monasterio, CNR/Regione Toscana, 56124 Pisa, Italy; walterl@ftgm.it; 4 National Agricultural and Food Centre Nitra, Hlohoveck’a 2, 95141 Lužianky, Slovakia; francesco.vizzarri@nppc.sk; 5 Crop Science Research Center, Scuola Superiore Sant’Anna, Piazza Martiri della Libertà 33, 56127 Pisa, Italy; 6 eCIER Research Group, Department of Biotechnology, Universidad Técnica del Norte, Av. 17 de Julio 5–21 y Gral. José María Córdova, Ibarra 100150, Ecuador; szarate@utn.edu.ec (S.Z.B.); casandoval@utn.edu.ec (C.S.); mecheverria@utn.edu.ec (C.E.)

**Keywords:** functional food, antioxidants, nutrition, green microalgae

## Abstract

Recently, green microalgae have gained importance due to their nutritional and bioactive compounds, which makes them some of the most promising and innovative functional foods. The aim of this study was to evaluate the chemical profile and the in vitro antioxidant, antimicrobial and antimutagenic activity of an aqueous extract of the green microalga *Ettlia pseudoalveolaris*, obtained from the freshwater lakes of the Ecuadorian Highlands. Human microvascular endothelial cells (HMEC-1) were used to determine the ability of the microalga to reduce the endothelial damage caused by hydrogen peroxide-induced oxidative stress. Furthermore, the eukaryotic system *Saccharomyces cerevisiae* was used to evaluate the possible cytotoxic, mutagenic and antimutagenic effect of *E. pseudoalveolaris*. The extract showed a notable antioxidant capacity and a moderate antibacterial activity mostly due to the high content in polyphenolic compounds. It is likely that the antioxidant compounds present in the extract were also responsible for the observed reduction in endothelial damage of HMEC-1 cells. An antimutagenic effect through a direct antioxidant mechanism was also found. Based on the results of in vitro assays, *E. pseudoalveolaris* proved to be a good source of bioactive compounds and antioxidant, antibacterial and antimutagenic capacities making it a potential functional food.

## 1. Introduction

In recent years, microalgae have become increasingly important in nutraceuticals and cosmetics due to the high content in carotenoids and other organic pigments. In addition, they contain various bioactive compounds such as β-carotene, astaxanthin, picocyanin and ω-3 polyunsaturated fatty acids that have potential pharmaceutical applications due to their anti-cancer, anti-aging, neuroprotective and antimicrobial effects. In fact, microalgae synthesize different types of high value-added compounds which can be exploited for different applications, especially in the food supplements sector [1]. Furthermore, besides the classic amino acids used for protein synthesis, microalgae are capable of producing the so-called mycosporine-like amino acids (MSAs), small metabolites produced by organisms usually living in marine environments whose synthesis is influenced by high exposure to sunlight [2]. In addition to their main, well-known function of sun protectors, these metabolites are antioxidants capable of stabilizing free radicals, thus protecting cells from reactive oxygen species (ROS) [3].

The oxidative damage caused by ROS at the level of lipids, proteins and nucleic acids can contribute to the onset of various chronic diseases such as cardiovascular disorders, atherosclerosis, cancer and aging. Epidemiological studies have shown an inverse association between fruit and vegetable intake and mortality from diseases related to aging such as, for example, coronary heart disease or cancer, which can also be attributed to their antioxidant activity. Since synthetic antioxidants, such as butylated hydroxytoluene (BHT) and butylated hydroxyanisole (BHA) have been shown to be toxic and carcinogenic in animal models, research is increasingly seeking to replace them with antioxidants of natural origin [4]. As a consequence, it is important to identify new sources of antioxidants of natural origin that are safe and economical. Due to the high content in bioactive compounds, microalgae are considered a very important source of natural antioxidant substances and it has been shown that the total content of phenolic compounds contributes to their antioxidant activity [5]. The main molecules identified within the microalgae are phloroglucinol, hydroxybenzoic and hydroxycinnamic acid-derived phenolic acids [6]. However, not all microalgae can be used as a natural source of antioxidants because of their great biodiversity, which implies a wide variety of by-products within them and a different yield depending on the species taken into account but also to other factors, such as cultivation methods and growth conditions [7].

Human Microvascular Endothelial Cells (HMEC)-1 is an immortalized cell line, transfected with the PBR-322-based plasmid containing the coding region for the Simian Virus 40 large T. Microcirculation has a relevant role in the genesis of target organ damage in various diseases such as diabetes, hypertension and dyslipidemia. Compared to large and medium-sized vessels, the microcirculatory system is the first to be involved in metabolic diseases. In addition, microcirculatory endothelial cells are the most numerous and representative of all endothelial cells of the body. Finally, this cell line facilitates the performance of many culture passages because the cells maintain the morphological, phenotypical and functional characteristics of normal human microvascular endothelial cells over time [8,9]. Thus, HMEC-1 cells represent an important experimental group in vitro models to study the preventive effect of green microalgae on endothelial disfunction. Oxidized low-density lipoprotein receptor 1 (LOX-1) is the major receptor for oxidized (Ox-)LDLs, which is able to bind and internalize by receptor mediated endocytosis [10]. It is an important marker of all cardiovascular risk factors; in fact, LOX-1 expression is upregulated in pathological conditions, such as hypertension, diabetes and hypercholesterolemia [11,12]. IL-6 is a well-known pro-inflammatory cytokine that is produced also by vessels and stimulates the inflammatory and auto-immune processes in many diseases, including atherosclerosis [13]. LOX-1 and IL-6 markers are increased in the presence of oxidative stress and inflammation in HMEC-1, thus resulting representative of endothelial dysfunction.

The antimicrobial activity of microalgae has been discovered through growth inhibition studies on bacteria and fungi responsible for different pathologies [14]. Polyphenols contained in microalgae have been identified as the main responsible for this activity by inhibiting nucleic acid synthesis, membrane cytoplasmic function, energy metabolism, and alteration of membrane permeability. Furthermore, polyphenols are able to attenuate the pathogenicity of microorganisms by reducing many virulence factors such as adhesion molecules, bacterial toxins and inhibition of biofilm formation [15].

As they are rich in essential nutrients, microalgae represent one of the main sources of food, especially in East Asia where, in particular, green ones have been used as food and supplements for hundreds of years. To date, microalgae are consumed all over the world for their remarkable nutritional value; among these, the most consumed are the green algae (*Chlorophyceae*), such as *Chlorella vulgaris* (chlorella), *Haematococcus pluvialis*, *Dunaliella salina*, and the cyanobacterium *Spirulina maxima* (spirulina), widely marketed and mainly used as supplements for human nutrition and as additives for animal feed [16].

The aim of the present study was to determine the fatty acids and polyphenolic composition, the antioxidant, antimicrobial and antimutagenic activity of an extract obtained from a green microalga from Ecuador, *Ettlia pseudoalveolaris*, in order to evaluate its possible use as a Novel Food [17]. To achieve this aim, different culture media were evaluated for microalgae biomass production, then, the extract was quantified for total and individual phenolic compounds and pigments; furthermore, it was used for performing in vitro tests for the determination of the antioxidant activity, among which is the in vitro evaluation of the potential effect on the reduction of lipid peroxidation in rat livers. The antibacterial activity was determined on four different strains of pathogenic bacteria. Furthermore, a study on microcirculatory endothelial cells (HMEC-1) was performed to evaluate the potential reduction of some markers of endothelial dysfunction (LOX-1 and IL-6), which are increased in a condition of oxidative stress and inflammation. The effect of the extract on the quantity of metabolites derived from nitric oxide (NO), which represents a very important biological messenger, was also observed. Finally, *Saccharomyces cerevisiae* yeast strain D7 was used as a model organism to determine the potential genotoxic and protective effect on oxidation-induced damage.

## 2. Materials and Methods

### 2.1. Strain, Cultivation Medium and Pre-Cultivation

*Ettlia pseudoalveolaris*, (T.R. Deason & H.C. Bold) J. Komarek (syn. Parietochloris pseduoalveolaris (T.R. Deason & H.C. Bold) Shin Watanabe and G.L. Floyd, (accession number OP136137) freshwater microalgae isolated in the Plant Biotechnology Laboratories of the North-Technical University (Universidad Técnica del Norte, Ibarra, Ecuador Available online: www.utn.edu.ec (accessed on 23 May 2023)) was used [18]. Cultivation samples were pre filtered in 0.2 µm pore size filters to concentrate biomass and cultivated in 250 mL Erlenmeyer Flasks with 150 mL of culture media. Incubation was carried out in an orbital shaker (300 rpm) at 26 °C with a light intensity of 50 µmol/m/s in two different photoperiods of 18:6 and 24:0 day:night cycle for strain adaptation and biomass production, respectively. 

Different culture media were assessed to determine the most suitable nutritional conditions in cell adaptation and production stages. They were based on different concentrations of KH_2_PO_4_, K_2_HPO_4_, as a phosphate and potassium sources; KNO_3_ as a nitrogen source, CaCl_2_·2H_2_O, FeSO_4_·7H_2_O, MgSO_4_·7H_2_O, MnSO_4_·H_2_O and CuSO_4_·5H_2_O as micronutrient source. In addition, the Murashige and Skoog medium (MS) based on 4.4 g/L of Murashige and Skoog (Duchefa Biochemie) with extra nitrogen source as KNO_3_ (3.0 g/L) and phosphate (0.1 g/L) was used. These cultures were performed in solid media with agar 12 g/L (Difco™ Technical Agar) and liquid media. Moreover, 0.4 g of NaHCO_3_ and 0.1 g of 4-(2-hydroxyethyl) piperazin-1-etranesulfonic acid (HEPES) as pH buffer [19] were added to all culture media. After adjusting cultures at pH 7, an autoclaving sterilization step was applied at 121 °C, 15 psi for 20 min. 

### 2.2. Experimental and Photobioreactor Setup

*E. pseudoalveolaris* biomass production was performed in a photobioreactor with a constant light intensity 100 ± 10 µmol/m/s and 24:0 day:night cycle. The working volume of 0.6 L was kept at 26 °C during cultivation. Mixing was made by aeration with a flow rate of 0.5 L/min of sterilized air. Photobioreactor was run in batch mode with enough nitrogen to avoid starvation. The photoreactor was inoculated with 0.1 g/L of biomass concentration.

Optical densitity (OD) was measured at 750 nm three times at day by duplicate in an UV-VIS spectophotometer (Jenway 6705 UV/Vis). At the same time, cell number was measured by Neubauer chamber [18].

### 2.3. Harvest and Sample Preparation

After cultivation, microalgae were harvested during the linear growth phase after 4 days of cultivation when a biomass concentration of 1.2 g/L was reached. The algae biomass was centrifuged at 4500 rpm for 10 min, washed twice with 0.5 M ammonium formate, and stored at −20 °C. Afterwards, samples were lyophilized for 24 h for further analyses [20].

### 2.4. Microalgae Extract Preparation

Microalgae sample underwent double extraction in 80% (*v*/*v*) solution of methanol and water for the analysis [21]. Briefly, 2.5 mL of 80% methanol were added to 250 mg of sample. After 2 h-stirring in the dark, the sample was centrifuged for 30 min at 4000 rpm at room temperature and the supernatant was collected; the pellet was used for further extraction with additional 2.5 mL of 80% methanol. The supernatants of the two extractions were pooled (final concentration of 50 mg/mL) and stored at −20 °C until use. Extractions were performed in triplicate. 

For antimicrobial, antimutagenic activities and evaluation of the preventive effect on H_2_O_2_-exposed HMEC-1 cells, the methanolic extract (50 mg/mL) was concentrated by SpeedVac (Thermo Fisher Scientific, Waltham, MA, USA) to obtain a complete evaporation of the solvent, since it is toxic for bacteria, yeasts and endothelial cells. The sample was then resuspended in water to obtain a concentration of 500 mg/mL. Before the tests, the solution was filtered with a 0.45 µm filter and, when necessary, diluted with sterile water.

### 2.5. Proximate and Fatty Acids Composition of Microalgae

Ground samples of *E. pseudoalveolaris* green algae (<1 mm) were analyzed for dry matter (DM, method 950.46), ash (method 920.153), lipid (method 920.39), crude protein (method 990.03) and crude fiber (method 978.10), according to the standard methods of the Association of Official Analytical Chemists [22]. 

Fatty acids (FAs) composition of *E. pseudoalveolaris* was determined by gas chromatography after extraction and methylation [23,24]. Flame-ionization detection (FID) gas chromatography analyses were performed using an Agilent 7890A chromatograph equipped with a chromatography column for fatty acid methyl esters (FAME) (60 m × 0.25 mm × 0.20 μm; Phenomenex, Torrance, CA, USA). The temperature program was the following: starting temperature of 160 °C for 2 min, which was then increased by 2 °C/min until the temperature reached 250 °C; the injector temperature was set at 250 °C and the detector temperature was 250 °C. The peaks were identified by comparison with the known standard mixture of FAs (Sigma Aldrich, St. Louis, MI, USA) and expressed as % of total identified FAs. 

### 2.6. Antioxidant Profiling of E. pseudoalveolaris Extract

#### 2.6.1. Bioactive Molecules Content 

Total phenols were determined by the *Folin-Ciocalteu* colorimetric method [25] and expressed as mg of gallic acid equivalents (GAE)/g dry weight (dw). Total flavonoids were quantified using the aluminum chloride colorimetric method and expressed as mg catechin equivalent (CE)/g dw [26]. Total flavonols were measured as previously described by Souid et al. [27] and expressed as mg quercetin equivalent (QE)/g dw. Total monometric anthocyanins were quantified using the differential pH spectrophotometric method described by Lee et al. [28] and expressed as mg cyanidin-3-glucoside (C3GE)/100 g dw. The pigments chlorophyll A and B were quantified using the method of Lichtenthaler et al. [29] and expressed as μg of ChlA and ChlB/g dw. 

#### 2.6.2. Phenolic Compounds Profiling by UHPLC-ESI-MS/MS Analysis

Selected known phenolic compounds were chosen for a quantitative analysis of the extract by UHPLC-ESI-MS/MS using a Sciex 5500 QTrap+ mass spectrometer (AB Sciex LLC, Framingham, MA, USA), equipped with a Turbo V ion-spray source and coupled to an ExionLC AC System custom made by Shimadzu (Shimadzu Corporation, Kyoto, Japan) which includes two ExionLC AC pumps, autosampler, controller, degasser and tray. MS/MS experiments were performed in the electrospray negative ion mode using nitrogen as collision gas. The operation source parameters were source type, turbospray; nebulizer gas (GS1) 70; turbo gas (GS2) 50; curtain gas (CUR) 10; temperature (TEM) 500 °C; Ionspray Voltage (IS) −4500 V, entrance potential (EP) 10 V. Compound parameters, declustering potential (DP), collision energy (CE), and collision cell exit potential (CXP) were adjusted for the specific Selected Reaction Monitoring (SRM) transition for any component. Analyses were performed in triplicate and results are expressed as µg/100 g dw.

#### 2.6.3. In Vitro Antioxidant Activity Assays

The antioxidant capacity of *E. pseudoalveolaris* extract was explored in vitro using a combination of fluorimetric and spectrophotometric methods, namely the oxygen radical absorbance capacity (ORAC) assay and the ferric reducing antioxidant power (FRAP) assay. The antioxidant capacity was quantified using the ORAC assay according to Ninfali et al. [30]. AAPH was used as peroxyl radical generator and fluorescein as a probe. Trolox was used as antioxidant standard. Results were expressed as ORAC units, namely μmol Trolox equivalents (TE)/g dw.

The total antioxidant power of *E. pseudoalveolaris* extract was determined using the FRAP assay, a colorimetric method based on the reduction of a ferric tripyridyltriazine complex to its ferrous form, as described by Colosimo et al. [31]. Data were translated into the FRAP value (µM) using a water solution of Fe (II) in the range of 100–2000 μM FeSO_4_ × 7H_2_O for calibration.

The ability of *E. pseudoalveolaris* to counteract lipid peroxidation, induced by the use of Fenton’s reagent, was evaluated on a pool of three liver samples from rats bred in Centre of Experimental Biomedicine at CNR (Pisa, Italy) at the end of an in vivo protocol (Protocol 65E5B.56) authorized by the Italian Ministry of Health (Authorization 67/2021-PR of 27/04/21). Free radical-mediated lipid peroxidation was determined by measuring the endogenous and stimulated accumulation of thiobarbituric acid reactive substance (TBARS) as previously described by Houng et al. [32]. Results were reported as IC50 (mg/mL) corresponding to the concentration of the extract capable of inhibiting the formation of TBARS by 50%, compared to the control. 

### 2.7. Endothelial Cell Culture and Treatments

Human microvascular endothelial cells (HMEC-1) obtained from Centre of Disease Control (Atlanta, GA, USA) were used to determine the ability of the microalga to reduce the endothelial damage caused by hydrogen peroxide (H_2_O_2_)-induced oxidative stress. Cell cultures were prepared and maintained as described by Lubrano and Balzan [33]. To evaluate the effect of *E. pseudoalveolaris* extract on cell viability, cells were incubated for 24 h with four different concentrations (6.25–12.5–25–50 μg/mL) of extract. The highest concentration non-toxic on cell viability, which in this case corresponded to 25 μg/mL, was used for this experiment to test the antioxidant power of an oxidizing substance H_2_O_2_ (50 μM). Four different plates of cell plates were prepared in triplicate: controls (C); H_2_O_2_ 50 μM (H_2_O_2_); *E. pseudoalveolaris* extract 25 μg/mL (E); H_2_O_2_ 50 μM + *E. pseudoalveolaris* extract 25 μg/mL (H_2_O_2_ + E). The plates were kept in an incubator at 37 °C for 24 h until the removal of the culture medium, which will be subjected to various essays. The adherent cells were then detached with trypsin/EDTA and resuspended in type II medium for cell count in a Burker chamber. The content of LOX-1 (Lectin-like Oxidized Low-Density Lipoprotein (LDL) Receptor 1), IL-6 (interleukin-6) and nitrites and nitrates present in the culture medium of the cells were determined as markers of cellular activation and dysfunction. 

The results of LOX-1 immunoenzymatic assay are expressed as pg of LOX-1 per 100,000 cells (pg LOX-1/100,000 cells); those of IL-6 immunoenzymatic assay as pg of IL-6 per 100,000 cells (pg IL-6/100,000 cells). Total nitrite (NO_2_^−^) and nitrate (NO_3_^−^) measurements are based on the Griess reaction: nitrates are converted into nitrites by the enzyme nitrate reductase and involves the bonding of NO_2_^−^ with sulfanilamide, forming a diazotate compound that, reacting with N-(1-naphthyl) ethylenediamine, generate a pink colored product whose absorbance was measured at 540 nm. All assays were performed in triplicate and results obtained were expressed as µmol/L of NO_2_^−^ + NO_3_^−^ per 100,000 cells (µM NO_2_^−^ + NO_3_^−^/100,000 cells).

### 2.8. Antibacterial Activity

The effect of sample extract on selected bacteria (*Escherichia coli*, *Salmonella enterica* ser. Typhimurium, *Staphylococcus aureus* and *Enterococcus faecalis)* growth was determined according to Pozzo et al. [34], with some modifications. The tested bacteria were cultured in Muller Hilton Broth (MHB) at 37 °C for 16 h and diluted to match the turbidity of 0.5 McFarland standard. Fifty microliters of bacterial suspensions (about 1–5 × 10^5^ CFU/mL) were added to 100 µL of MHB and to 100 µL of extract (0.0031, 0.31, 6.25 and 50 mg/mL) in a 96-well plate. A negative control was included on each microplate. The plates were incubated at 37 °C for 24 h. Afterwards, the optical density (OD) at 630 nm was determined by a microplate reader (Eti-System fast reader Sorin Biomedica, Modena, Italy). The percentage of growth inhibition was calculated as follows:

% *growth inhibition* = 100 − (*ODS*/*ODC*) × 100 (1)

where *ODS* is the optical density of the sample and *ODC* is the optical density of the negative control.

### 2.9. Short Term Assays in Yeast Cells

Yeast cells (*Saccharomyces cerevisiae* D7 strain) from a standard culture were incubated in liquid medium containing 2% glucose under shaking (30 °C). There are several experimental methodologies for carrying out genotoxicity tests in yeast *S. cerevisiae* to evaluate the potential effect of a compound; in this study logarithmic phase cells were employed because they are metabolically active [35]. About 15 × 10^5^ cells/100 mL of liquid medium were incubated over-night under shaking (30 °C) until logarithmic phase was reached (70–90 × 10^6^ cells/mL), with or without *E. pseudoalveolaris* extract. The use of the diploid strain D7 allows the simultaneous determination of toxicity, mitotic gene conversion (GC), and point reverse mutation (PM) [36]. H_2_O_2_ (4 mM) was used as mutagen agent because it causes oxidative stress in cells; H_2_O_2_ is reduced by ions metals through the Fenton reaction with the consequent production of hydroxyl radicals [37]. Before setting up antimutagenesis assay for testing the potential protective effect of the microalga, preliminary tests were performed to determine the genotoxicity (survival and mutagenesis) of *S. cerevisiae* cells.

Two different experimental procedures were followed for both genotoxicity and antimutagenesis assays:

(1) *Growth assay: E. pseudoalveolaris* extract (50, 100, 200, and 400 μg/mL) was added in the flasks during growth until reaching the logarithmic phase; then H_2_O_2_ was added and the cultures were incubated for 90 min under shaking (30 °C).
(2) *Incubation assay*: logarithmic phase cells were incubated both with *E. pseudoalveolaris* extract (50, 100, 200, and 400 μg/mL) and H_2_O_2_ for 90 min, under shaking (30 °C).

### 2.10. Statistical Analysis

XLSTAT Version 2016 statistical software was used for statistical analyses. The results are presented as the mean (*n* = 3) value ± standard deviation (SD) and analyzed through a one-way analysis of variance (ANOVA) and Tukey post-hoc with significance at *p* ≤ 0.05.

## 3. Results and Discussion

### 3.1. Cultivation Conditions 

Microalgae growth in different solid culture media were not statistically different (*p* = 0.1801). In contrast, the cultivation in liquid media based on MS supplemented with nitrogen and phosphate demonstrated the highest microalgae growth in batch operation. Microalgae culture grew from 0.1 g/L to 1.2 g/L after 83 h. Viable cell concentration increased until 13.8 × 10^6^ cell/mL at the final exponential growth phase. The pH of the cultures rose rapidly from 7.2 to 8.3 after four days and it was remained during cultivation. Moreover, culture temperatures ranged from lows of 25 °C ± 0.7 °C to highs of 26 °C ± 0.5 °C during the experiments.

### 3.2. Proximate and Fatty Acids Composition

The proximate composition and FAs profile of *E. pseudoalveolaris* are presented in Table 1. The FAs composition of *E. pseudoalveolaris* can vary depending on several factors, such as the growth phase, cultivation conditions, and extraction method. Our results showed that *E. pseudoalveolaris* contained significant amounts of saturated fatty acids (SFAs), such as palmitic acid (C16:0), which is by far the most representative (35.94 ± 0.11%), myristic (C14:0) and stearic (C18:0) acid, which have various industrial applications. In example, a good quality biodiesel should be rich in palmitic and stearic acids: a high percentage of SFAs favors better viscosity (flow properties) and the applicability of biodiesel at low temperatures [38]. Additionally, in a very recent FAs profile analysis of *Chlorella vulgaris*, in each of the four different strains examined, palmitic acid resulted the most representative among the SFAs [39]. Another study on other green microalgal strains (*T. obliquus* and *D. abundans*) showed that SFAs represent most of total fatty acid methyl esters profile, confirming that many microalgae are rich in this type of FAs [40]. Additionally, *E. pseudoalveolaris* proved to be rich in polyunsaturated fatty acids (PUFAs), particularly in alpha-linoleic acid (ALA) (29.28 ± 0.12%) and linoleic acid (LA) (14.95 ± 0.06%), in accordance with what observed in *Chlorella vulgaris* by Mauricio et al. [39]. As essential ω-3 FAs, ALA and LA play a crucial role for human health and must be obtained from dietary sources. In fact, humans and other mammals are not capable to de novo synthesize ω-3 and ω-6 PUFAs, due to the absence of Δ12 and Δ15 dehydrogenases. The synthetic pathways of ω-3 and ω-6 PUFAs share common chain-elongation and desaturases enzymes, determining a metabolic competition process. Moreover, the essential fatty acid ALA serves as a precursor to ω-3 PUFAs such as EPA and DHA. Thus, supplementing these precursors can help also to increase EPA or DHA levels, with a more comprehensive supplementing effect [41]. While ALA is typically found in plant-based sources such as flaxseed and chia seeds, some microalgae are also rich in this nutrient resulting a crucial source of PUFAs, especially considering that algae are the original source of ω-3 PUFAs in fish oil. A study by De Pauw et al. [42] identified several microalgal species with high levels of ALA, including *Nannochloropsis*, *Tetraselmis*, and *Isochrysis*. Another green microalga, *Chlamydomonas reinhardtii*, showed a FAs profile similar to those of *E. pseudoalveolaris*, being palmitic acids, among SFAs, and ALA, among PUFAs, the most represented FAs [43]. A study by Nicoletti et al. [44] found that the green microalga *Thorsmoerkia curvula* also contained significant amounts of ALA, thus confirming the potential of green microalgae as a viable source of ALA with various applications in food, nutraceuticals and animal feed. Although to a lesser extent then PUFAs, monounsaturated fatty acids (MUFAs) were also present in the FAs profile of *Ettlia*, with palmitoleic (3.35 ± 0.03%) and oleic acid (3.28 ± 0.02%) as the most represented. MUFAs consumption has been associated with decreased low-density lipoprotein (LDL), increased high-density lipoprotein (HDL) cholesterol and even with the regulation of body weight [45]. Overall, the unique chemical and fatty acid compositions of *E. pseudoalveolaris* makes it a valuable resource for a range of biotechnological and industrial applications.

### 3.3. Antioxidant Profiling of E. pseudoalveolaris Extract

#### 3.3.1. Phytochemical Characterization 

The freeze-dried microalgae methanolic extract showed a total phenols content of 9.04 ± 0.80 mg GAE/g dw, a flavonoids content of 13.51 ± 1.11 mg CE/g dw, a flavonols content of 4.17 ± 0.58 mg QE/g dw, and a content of anthocyanins equal to 3.39 ± 0.61 mg C3GE/100 g dw (Table 2). The amount of the pigments chlorophyll A and B was 2.1 and 1.9 μg/g dw, respectively (Table 2). To compare these results with those performed on other green microalga, it is necessary to consider that the composition of the antioxidants is strongly influenced by the growth conditions of the microalgae (availability of nutrients, temperatures, stress factors, etc.), the solvents used for the extraction, and, obviously, the different species taken into account [5,46]. The value obtained for total phenols is consistent with the results reported in the study by Maadane et al. [47] in which several species of green microalgae were analyzed and whose content, determined with the Folin-Ciocȃlteu method, varied from 8.10 (*Chlorella* sp.) to 32 mg GAE/g (*Nannochloropsis gaditana*). The polyphenolic content of our extract of *E. pseudoalveolaris* was higher than those obtained by Goiris et al. [5], in which biomass from 32 species of microalgae were analysed with an average of total polyphenols was equal to 2.11 mg GAE/g dw. Up to now, there are only a few studies in literature that examine pigments and subcategories of polyphenols of microalgae. These studies confirm that the quantity of these compounds can vary significantly depending on the species and the conditions under which they are grown [48]. In a recent study by Tiong et al. [49], the concentration of flavonoids was measured in five different species of microalgae and found to range from 14 to 34.7 mg of CE/g dw, which is similar to the level found in *E. pseudoalveolaris* extract. The levels of chlorophyll A and B found in *E. pseudoalveolaris* extract (6.99 μg/mL and 6.27 μg/mL, respectively) are comparable to those reported by Oo Y.Y.N. et al. [50] in the *Chlorella* sp. and *Nannochloropsis* sp. species, ranging from 4.33 to 21.24 μg/mL.

The phytochemical profile of *E. pseudoalveolaris* was further investigated by UHPLC-ESI-MS/MS analysis to determine the individual phenolic constituents (Table 3). Twenty-eight compounds were identified in the extract. It is important to note that polyphenols extraction efficiency and, consequently, their concentration are dependent on the polarity of the solvent used, since it determines the solubility of polyphenols based on their structure. Consequently, the reproducibility of these data is strictly dependent on the procedure used. By far the most representative phenolic compound found in our extract was verbascoside (3054.48 ± 31.16 µg/100 g dw), which is widely known for its anti-inflammatory and antimicrobial properties, notably against *Staphylococcus aureus*. In particular, this polyphenol is able to induce a lethal effect on bacteria by affecting protein synthesis and inhibiting leucine incorporation [51]. Moreover, it is a protein kinase C inhibitor. Although some in vitro genotoxicity of verbascoside has been reported on human lymphocytes with an involvement of PARP-1 and p53 proteins, subsequent in vivo tests reported no genotoxicity for high dosage oral administration [52].

#### 3.3.2. In Vitro Antioxidant Properties

To evaluate the antioxidant activity of the methanolic extract, we performed three different tests. With the ORAC assay we measured the ability of the extract to inhibit the oxidative degradation of a fluorescent molecule caused by the attack of peroxyl radicals (ROO). The value of 82.4 μmol TE/g dw obtained in the ORAC assay was slightly higher compared to the results obtained in the 2019 study by Banskota et al. [53] for nine different microalgal species, which ranged from 21.21 to 69.48 μmol TE/g. The inhibition efficiency of lipid peroxidation was tested by TBARS and the ability to reduce Fe^3+^ to Fe^2+^ by FRAP assay. In the study conducted by Habashy et al. [54] on the well-known green microalga Chlorella vulgaris the TBARS assay showed an IC50 of about 0.025 mg/mL, considered an indication of a high activity against lipid peroxidation. The results obtained on the *E. pseudoalveolaris* extract showed a good reducing power (11.1 ± 0.7 mg FE^2+^/g) and a lipid peroxidation reduction activity (IC50 equal to 7.78 ± 1.54 mg/mL), demonstrating a possible role in the prevention of oxidative damage to biomolecules (Table 2).

### 3.4. Effects on HMEC-1 Cells Treated with H_2_O_2_

**Figure 1:**
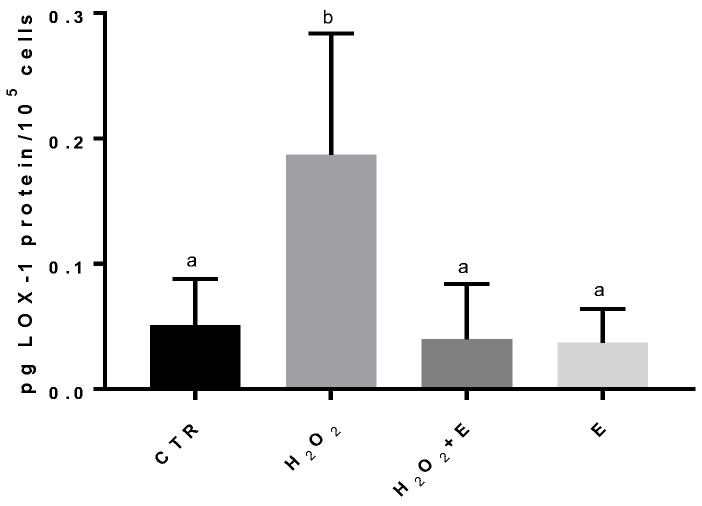
LOX-1 protein levels in HMEC-1 cells by immunoenzymatic assay, expressed as pg of LOX-1 per 100,000 cells, measured in CTR, H_2_O_2_, H_2_O_2_ + E (*E. pseudoalveolaris*) and E cells. Data represent the mean ± SD (bars). a, b: values significantly different by one way ANOVA-test, *p* = 0.0359.

HMEC-1 cells treatment with H_2_O_2_ determined a significant increase in the amount of LOX-1 protein in comparison to control cells, passing from 0.051 ± 0.017 pg of LOX-1/10^5^ cells in the case of the control to 0.187 ± 0.047 pg of LOX-1/10^5^ cells in the case of cells subjected to oxidative stress. When the cells were treated with both *E. pseudoalveolaris* and H_2_O_2_, we did not observe any significant increase in LOX-1, which remained equal to control cells (0.040 ± 0.014 pg/10^5^ cells), as it happened in the case of treatment with *E. pseudoalveolaris* only (0.037 ± 0.010 pg/10^5^ cells) (Figure 1).

**Figure 2:**
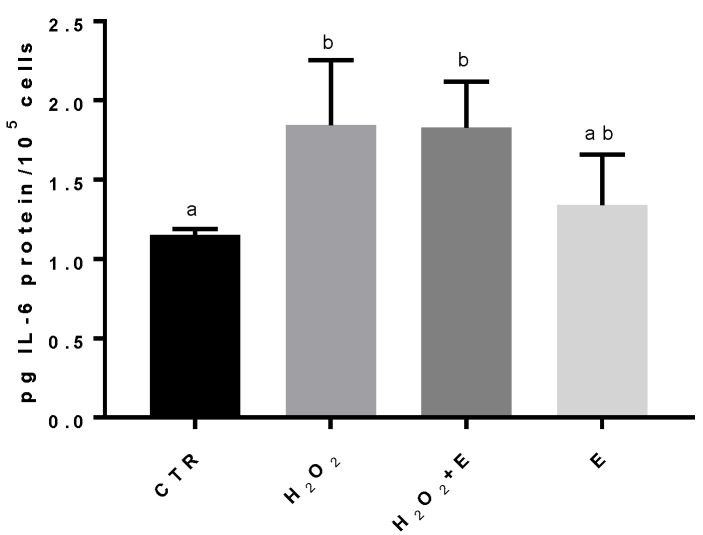
IL-6 protein levels in HMEC-1 cells by immunoenzymatic assay, expressed as pg of IL-6 per 100,000 cells, measured in CTR, H_2_O_2_, H_2_O_2_ + E (*E. pseudoalveolaris*) and E cells. Data represent the mean ± SD (bars). a, b: values significantly different by one way ANOVA-test, *p* = 0.0478.

Cells treated with H_2_O_2_ showed a significant increase in the amount of IL-6, compared to control cells, with protein levels increasing from 1.150 ± 0.044 pg/10^5^ cells in the case of the control to 1.845 ± 0.411 pg/10^5^ cells in the case of H_2_O_2_-treated cells. The same significant increase also occurs in the case of cells treated with *E. pseudoalveolaris* and H_2_O_2_ (1.829 ± 0.296 pg/10^5^ cells). In contrast, we did not observe any significant increase in IL-6 level in cells treated with *E. pseudoalveolaris* only (1.341 ± 0.323) compared to the control (Figure 2).

The statistically significant increase in both LOX-1 and IL-6 content observed with 50 μM H_2_O_2_ treatment is a direct consequence of the oxidative stress caused by free radicals in endothelial cells, generating a condition of “endothelial dysfunction” through different mechanisms. In fact, oxidative stress generated by free radicals results in a direct increase in LOX-1 expression in endothelial cells [55] thus leading to the activation of a series of metabolic pathways, including those of NF-kB, which in turn stimulate the production of inflammatory cytokines, including IL-6 [56], whose secretion is usually upregulated in response to inflammation and oxidative stress. LOX-1 is also responsible of the intracellular synthesis of additional ROS via the activation of the enzyme NADPH oxidase [57]. Furthermore, the increase in IL-6 leads to an increased expression of the oxidized LDL receptor (LDLr), thus triggering a pro-inflammatory “vicious circle” [58]. It has been shown that even small changes in blood IL-6 levels are able to increase the expression of the LDLr and, consequently, reduce LDL concentration in plasma. Similarly, it was observed that IL-6 stimulation of endothelial cells determined an increase in LOX-1 levels, thereby contributing to the progression of endothelial dysfunction [33].

When the cells were treated with both *E. pseudoalveolaris* and H_2_O_2_, we observed a significant decrease in LOX-1, but not in IL-6 level (Figure 1 and Figure 2, respectively). An extended incubation period of the cells with the extract has been scheduled to investigate the potential impact of prolonged exposure on the inflammatory pathway.

The incubation with the microalga alone confirmed that the concentration used (25 ug/mL) did not have a toxic effect on HMEC-1 cells, since the values of LOX-1 and IL-6 were not significantly different from those of control cells.

**Figure 3:**
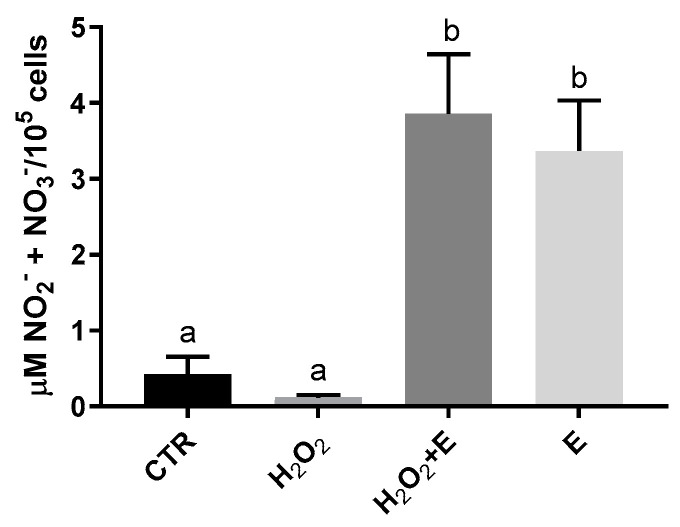
Nitrites and nitrates levels in HMEC-1 cells based on Griess reaction, expressed as µM NO_2_^−^ + NO_3_^−^/100,000 cells, measured in CTR, H_2_O_2_, H_2_O_2_ + E (*E. pseudoalveolaris*) and E cells. Data represent the mean ± SD (bars). a, b: values significantly different by one way ANOVA-test, *p* < 0.0001.

The content of nitrites and nitrates decreased, although not significantly, in HMEC-1 cells treated with H_2_O_2_ (0.119 ± 0.032 µM/10^5^ cells), compared to the control (0.421 ± 0.234 µM/10^5^ cells) (Figure 3). Both in the case of treatment with *E. pseudoalveolaris* + hydrogen peroxide, and in the case of treatment with *E. pseudoalveolaris* alone, a statistically significant increase of nitrites and nitrates was observed (3.855 ± 0.790 and 3.373 ± 0.660 µM of NO_2_^−^ + NO_3_^−^/100,000 cells, respectively) (Figure 3).

The decrease observed in H_2_O_2_-treated cells could be due to the activation and damage of the nitric oxide synthases (NOS) enzyme induced by free radicals which leads to a reduction in production of NO by endothelial cells, as demonstrated in other studies [55]. An increase in ROS also results in an overexpression of LOX-1 which in turn determines a decrease in Endothelial NOS (eNOS) expression. Furthermore, a direct action of free radicals on NO molecule causing its denaturation could also contribute to this reduction [33].

On the contrary, the significant increase in NO in *E. pseudoalveolaris*-treated cells both in the presence and absence of H_2_O_2_, may be due to the fact that the microalga, through its antioxidant action, is capable to neutralize free radicals and promote the activity of the enzyme responsible for the production of NO. In fact, several studies show that antioxidant substances are responsible for this effect, thus determining a positive effect on the state of the endothelium [59].

### 3.5. Antibacterial Activity

**Figure 4:**
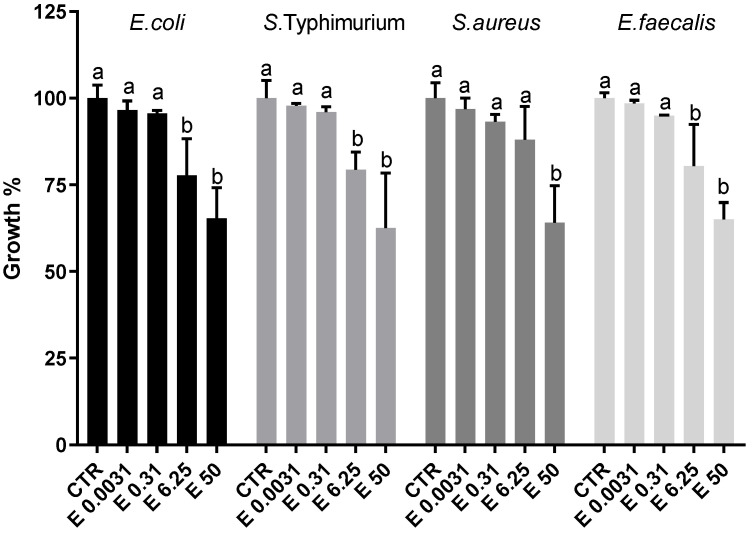
Growth percentage of Gram-negative (*E. coli* and *S. typhimurium*) and Gram-positive (*S. auresus* and *E. faecalis*) bacterial strains at different concentrations (0, 0.0031, 0.31, 6.25 and 50 mg/mL) of *E. pseudoalveolaris* (E) extract. Data represent the mean ± SD (bars). a, b: values significantly different by one way ANOVA-test, *p* = 0.0003 (*E. coli*); *p* = 0.0007 (*S. typhimurium*); *p* = 0.0006 (*S. aureus*); *p* < 0.0001 (*E. faecalis*).

Antibacterial activity of *E. pseudoalveolaris* was reported in terms of growth percentage for each different bacterial strain grown with different concentrations of the extract, in comparison to the respective control (growth% = 100 ± SD), and reported in Figure 4. For each bacterial strain, the concentrations of the extract used in the experiment were 50, 6.25, 0.31 and 0.0031 mg/mL. In the case of Gram-negative bacteria, a significant reduction in bacterial growth was observed for both *E. coli* and *S. typhimurium* at the concentrations of 50 and 6.25 mg/mL (65.33 ± 8.84% at the concentration of 50 mg/mL and 77.72 ± 10.62% at the concentration of 6.25 mg/mL for *E. coli*, respectively and 62.53 ± 15.79% at the concentration of 50 mg/mL and 79.38 ± 5.06% at a concentration of 6.25% for *S. typhimurium* , respectively). At lower concentrations, no significant reduction in the percentage of bacterial growth was observed for either strain, in comparison to controls. Regarding Gram-positive, a significant reduction of growth% was observed for *E. faecalis* at the concentrations of 50 and 6.25 mg/mL (65.08 ± 4.80% and 80.46 ± 11.91%) while in the case of *S. aureus* the reduction was significant at the concentration of 50 mg/mL only (64.07 ± 10.66%). For all bacterial strains considered, the lowest concentrations of the extract (0.31 and 0.0031 mg/mL) have no effect on bacterial growth (Figure 4).

Several green algae, such as *Desmococcus olivaceous*, *Chlorococcum humicola*, *Chlorella vulgaris*, *Ulva fasciata*, *Enteromorpha intestinalis* have been studied for antibacterial activity in view of looking for new antimicrobial agents of natural origin [21,60]. In fact, microalgae are able to produce a large number of biologically active secondary metabolites that act as a “chemical defense” against herbivores, allowing them to compete for space and nutrients. To these molecules have been attributed bacteriostatic, bactericidal, antifungal, antiviral, anti-inflammatory and antitumor activities [21,61]. Several species of green microalgae have been shown to exhibit in vitro antibacterial activity against Gram-positive and Gram-negative bacteria. Strains of *Scenedesmus* (*S. obliquus* and *S. quadricauda*) have been demonstrated to exhibit antibacterial activity against *S. aureus*, *E. coli*, *Pseudomonas aeruginosa* and *Bacillus subtilis* [14,62]. Bhagavathy et al. [63] reported that purified pigments (carotenoids and chlorophyll) extracted with different organic solvents from the green microalga *Chlorococcum humicola* were capable of inhibiting the growth of *E. coli*, *S. typhimurium*, *Klebsiella pneumoniae*, *Vibrio cholera*, *P. aeruginosa*, *S. aurues* and *B. subtilis*. Furthermore, in a study by Ghasemi et al. [64], aqueous, methanolic and hexanol extracts of *Chlorella vulgaris* showed a growth inhibitory effect against *S. epidermidis*, *S. aureus* and *S. typhimurium*. For the first time, in this study, the antimicrobial activity of the methanolic extract of *E. pseudoalveolaris* was investigated; we observed a significant decrease in the growth of four different strains of pathogenic bacteria in the presence of the *E. pseudoalveolaris* extract at a concentration of 50 mg/mL. The extract did not show differences in the ability to inhibit the growth of Gram-positive or Gram-negative bacteria as the final growth percentage is very similar for the four strains. In addition, all bacteria show a significant decrease in growth percentage at the concentration of 6.25 mg/mL, with the exception of *S. aureus* (Figure 4). The major responsibility for this antimicrobial ability could be attributable to verbascoside, by far the most representative among the phenolic compounds found in our extract (Table 3), which is well-known for its antimicrobial activity [51]. As well as in the cases illustrated above, the results obtained in the present study show that *E. pseudoalveolaris* has a moderate antimicrobial activity, thus confirming that green microalgae are a good source of antibacterial substances, with possible applications in the pharmaceutical industry [65].

### 3.6. Short Term Tests in Yeast Saccharomyces cerevisiae 

#### 3.6.1. Genotoxicity Assays

**Figure 5:**
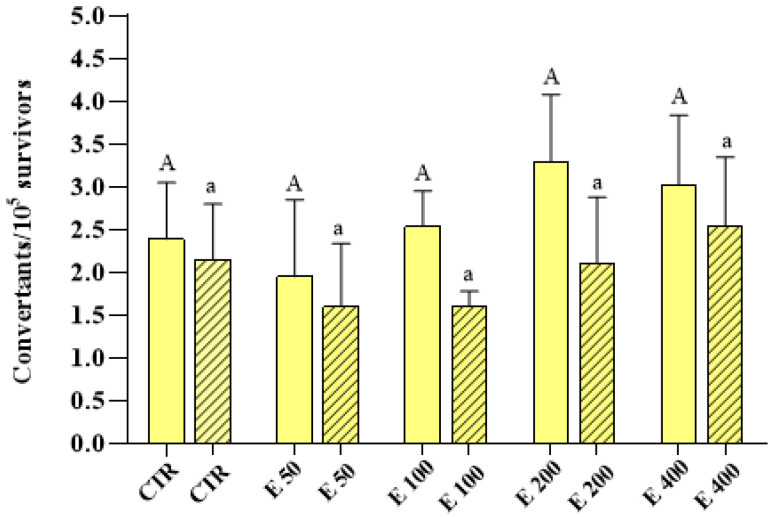
Mitotic gene conversion (GC) frequency on the D7 strain of yeast *S. cerevisiae* incubated with different concentrations (0, 50, 100, 200 and 400 μg/mL) of *E. pseudoalveolaris* (E) extract, added during cell growth (*growth assay*, empty bar) or after cell growth (*incubation assay*, striped bar). Results are reported as convertants/10^5^ survivors. Data represent the mean ± SD (bars). Capital letters are used for experimental mode (1), A, a: values significantly different by one way ANOVA-test.

**Figure 6:**
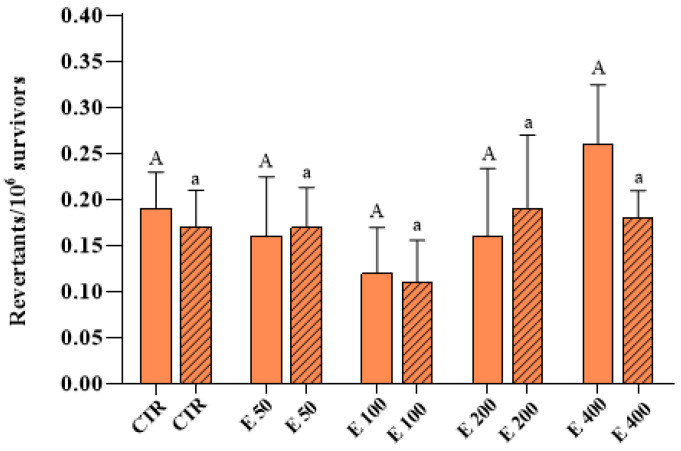
Point reverse mutation (PM) frequency on the D7 strain of yeast *S. cerevisiae* incubated with different concentrations (0, 50, 100, 200 and 400 μg/mL) of *E. pseudoalveolaris* (E) extract, added during cell growth (*growth assay*, empty bar) or after cell growth (*incubation assay*, striped bar). Results are reported as revertants/10^6^ survivors. Data represent the mean ± SD (bars). Capital letters are used for experimental mode (1), A, a: values significantly different by one way ANOVA-test.

Survival was calculated as a percentage of surviving cells with the respect to control cells; no statistically significant decrease was observed at all the concentrations tested, compared to the control (data not shown). Regarding mutagenesis tests, both in the experimental mode in which the microalga was incubated during cell growth (*growth assay*) and in the one in which it was incubated after the achievement of the logarithmic phase (*incubation assay*), the extract did not induce any significant increases in GC or PM frequencies, for all the concentrations used, compared to the control (Figure 5 and Figure 6, respectively).

#### 3.6.2. Antimutagenic Assay

**Figure 7:**
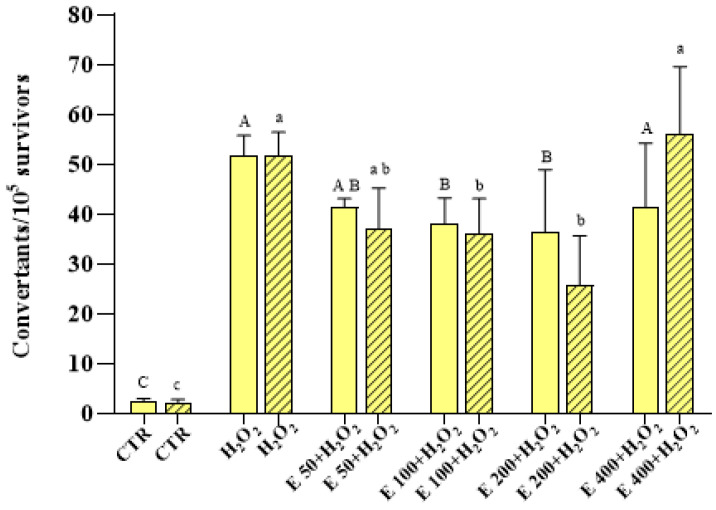
Mitotic gene conversion (GC) frequency on the D7 strain of yeast *S. cerevisiae* incubated with 4 mM H_2_O_2_ and different concentrations (0, 50, 100, 200 and 400 μg/mL) of *E. pseudoalveolaris* (E) extract, added during cell growth (*growth assay*, empty bar) or after cell growth (*incubation assay,* striped bar), compared to untreated cells (CTR). Results are reported as convertants/10^5^ survivors. Data represent the mean ± SD (bars). Capital letters are used for experimental mode (1), A, B, C; a, b, c: values significantly different by one way ANOVA-test, *p* = 0.001.

**Figure 8:**
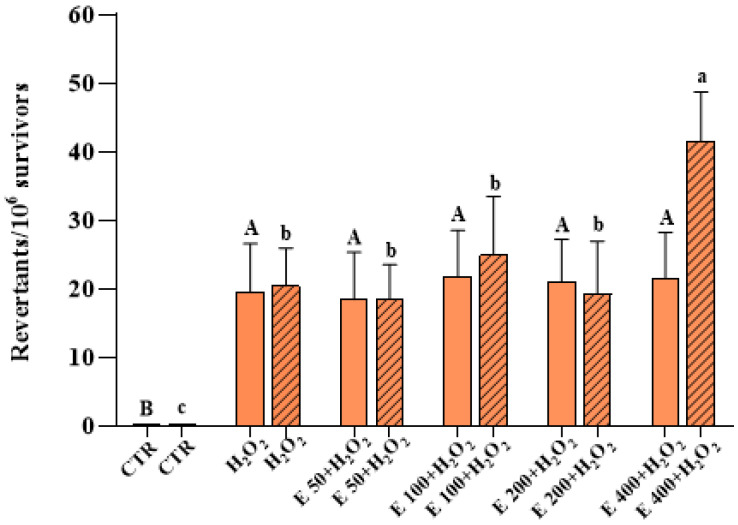
Point reverse mutation (PM) frequency on the D7 strain of yeast *S. cerevisiae* incubated with 4 mM H_2_O_2_ and different concentrations (0, 50, 100, 200 and 400 μg/mL) of *E. pseudoalveolaris* (E) extract, added during cell growth (*growth assay*, empty bar) or after cell growth (*incubation assay*, striped bar), compared to untreated cells (CTR). Results are reported as revertants/10^6^ survivors. Data represent the mean ± SD (bars). Capital letters are used for experimental mode (1), A, B, a, b, c: values significantly different by one way ANOVA-test, *p* = 0.0235.

The results, reported in Figure 7 and Figure 8, show that the oxidizing substance determined a significant increase in both the frequency of GC and PM, respectively, compared to the control. In fact, the frequency of the GC increased from 2.39 ± 0.66 convertants/10^5^ to 51.56 ± 4.21 convertants/10^5^ survivors in the case of (1) *growth assay*, and from 2.15 ± 0.65 to 51.62 ± 4.85 in the case of (2) *incubation assay* (Figure 7). Moreover, the frequency of PM passes from 0.19 ± 0.04 to 19.53 ± 7.08 revertants/10^6^ survivors in the experimental mode (1), and from 0.17 ± 0.04 to 20.44 ± 5.52 in the mode (2) (Figure 8). A statistically significant decrease in the frequency of GC was observed in cells treated with 100 and 200 μg/mL of *E. pseudoalveolaris* extract, for both the experimental methods. A decrease, although not significant, was observed also for 50 μg/mL, while no differences were observed for 400 μg/mL, in comparison to the control (Figure 7). The extract did not cause any statistically significant change in the frequency of PM induced by the oxidizing agent for any of the concentrations used, except for 400 μg/mL using experimental mode (2), in which the frequency of the MP increased from 20.44 ± 5.52 of the cells treated with H_2_O_2_ only to 41.58 ± 7, 20 revertants/10^6^ survivors (Figure 8).

A large number of methods have been developed for determining genotoxicity in different genetic models; the mutagenicity test in *S. cerevisiae* is one of the standard protocols that the United States Environmental Protection Agency (U.S. EPA) recommends in evaluating the mutagenic potential of a substance. The test in the *S. cerevisiae* D7 strain has been widely validated and is universally accepted by the regulatory authorities (Official Gazette of the European Union L. 142/201 of 31.5.2008). Several scientific studies have shown evidence that the yeast *S. cerevisiae* has a strong homology with the human genome allowing it to be used as a model cellular system in cytotoxicity studies [66]. In fact, different studies have used the D7 strain for the evaluation of the mutagenic and antimutagenic effects of various substances of natural or synthetic origin [35]. In the literature there are no studies that have evaluated the genotoxic and antimutagenic activity of *E. pseudoalveolaris*. In a 2018 study, the toxicity of the green alga *Chlamydomonas reinharditii* was evaluated using some strains of the bacterium *S.* Typhimurium and not any mutagenic effect was highlighted [67]. In the present study, we used logarithmic phase cells because of their permeability and active metabolism. *E. pseudoalveolaris* at four different concentrations did not cause cytotoxicity and mutagenesis. Antimutagenesis experiments show that not all the concentrations used were capable of reducing the oxidative cell damage caused by H_2_O_2_. Regarding GC, under our experimental conditions, the concentration of 50 μg/mL resulted too low to be able to contrast the high increase (about 25 times) in this genetic event caused by hydrogen peroxide. On the other hand, the slight, although not significant, increase observed for the highest concentration (400 μg/mL) can be explained by the similar increase observed for the same concentration, compared to the control, in the preliminary mutagenesis assay, which may be the cause of such “pejorative” effect (Figure 7). For what concerns PM, our results show that the green alga was not effective in reducing the frequency of this event (Figure 8). It is likely that *E. pseudoalveolaris* does not have any specific effect on this event, which occurs more rarely than the CG. The antioxidant substances present in the extract and deriving from the microalgae protect from GC with both a preventive (experimental mode (1)) and a direct (mode (2)) mechanism.

## 4. Conclusions

To our knowledge, this is the first study characterizing the green microalgae *E. pseudoalveolaris* for chemical composition and antioxidant, antibacterial and antimutagen activities. Based on the obtained results, we can assert that this microalga has a good amount in phenolic compounds, which plays a crucial role for the observed antioxidant activity. Microcirculation plays a significant role in the pathogenesis of target organ damage in diverse diseases, including diabetes, hypertension, and dyslipidemia. In metabolic disorders, the microcirculatory system is the initial site of involvement, preceding larger vessels. Moreover, microcirculatory endothelial cells are the predominant and representative cell type among all endothelial cells in the body. Consequently, microvascular endothelial cells HMEC-1 serve as a crucial in vitro experimental model to prove the protective effect of the bioactive substances-rich extract of the green microalga on the endothelium. As a matter of fact, *E. pseudoalveolaris* determined a significant reduction in LOX-1 and increase in NO production following the oxidative insult due to H_2_O_2_ treatment. In addition to the antioxidant activity, the antibacterial and antimutagenic activities also are attributable to the secondary metabolites present in *E. pseudoalveolaris*. In fact, a significant reduction in the growth of several Gram-positive and Gram-negative pathogenic strains and a reduction in H_2_O_2_-induced genotoxicity in *S. cerevisiae* yeast cells were shown in our experiments. Furthermore, this microalga resulted rich in PUFAs, particularly in ALA and LA, whose balanced intake is crucial for human health, since they represent essential ω-3 FAs that must be taken from diet.

All in all, this study confirms that *E. pseudoalveolaris* represents a possible new source of bioactive substances to be used in the nutraceutical field, due to its antioxidant, antibacterial and antimutagenic actions. Further analyses could be performed in order to broaden the knowledge regarding the effect on endothelial cells using an in vivo model to confirm the molecular interactions that have been highlighted in the in vitro HMEC-1 cell model. A more detailed investigation aimed to isolate the substances that mostly contribute to the antibacterial activity could also allow a possible use of *E. pseudoalveolaris* as a source of natural antibiotics. Finally, to support the results obtained from our experiments on *S. cerevisiae*, from which no cytotoxic and mutagenic effects emerged, it could be appropriate to perform further toxicity tests to confirm the safety of *E. pseudoalveolaris* as a possible ingredient for human nutrition.

## Figures and Tables

**Table 1:** Composition expressed as fraction on dry weight (% dw), and fatty acids (FAs) profile expressed as % of identified fatty acids of *E. pseudoalveolaris*.

Reference Compound	*E. pseudoalveolaris*
Moisture	63.15 ± 0.03
Ash	22.44 ± 0.09
Lipid	0.99 ± 0.05
Crude protein	8.31 ± 0.12
Crude fiber	5.11 ± 0.07
SFAs	
C14:0	5.17 ± 0.04
C15:0	0.37 ± 0.01
C16:0	35.94 ± 0.11
C18:0	2.41 ± 0.02
C20:0	0.86 ± 0.00
MUFAs	
C14:1	0.47 ± 0.00
C16:1-n7	3.35 ± 0.03
C18:1-n9	3.28 ± 0.02
PUFAs	
C18:2-n6 (LA)	14.95 ± 0.06
C18:3-n3 (ALA)	29.28 ± 0.12
C20:4-n6 (AA)	2.04 ± 0.02
C22:5-n3 (DPA)	1.88 ± 0.01

SFAs: saturated FAs; MUFAs: monounsaturated FAs; PUFAs: polyunsaturated FAs; LA: linoleic acid; ALA: alpha-linoleic acid; AA: arachidonic acid; DPA: docosapentaenoic acid. Values are reported as means ± s.d.

**Table 2:** Bioactive compounds and in vitro antioxidant activity of *E. pseudoalveolaris*.

		*E. pseudoalveolaris*
Bioactive compounds	Total polyphenols (mg GAE/g dw)	9.04 ± 0.80
	Flavonoids (mg CE/g dw)	13.51 ± 1.11
	Flavonols (mg QE/g dw)	4.17 ± 0.58
	Anthocyanins (mg C3GE/100 g dw)	3.39 ± 0.61
Pigments	Chlorophyll A (μg ChlA/g dw)	2.09 ± 0.02
	Chlorophyll B (μg ChlB/g dw)	1.88 ± 0.03
Antioxidant activity	ORAC (µmol TE/g dw)	82.38 ± 5.86
	FRAP (mg FE^2+^/g dw)	11.11 ± 0.73
	TBARS (IC50 = mg/mL)	7.78 ± 1.54

Values are reported as means ± s.d.

**Table 3:** Selected reaction monitoring (SRM) transitions and the corresponding phenolic compounds parameters and concentrations of *E. pseudoalveolaris* by UHPLC-ESI-MS/MS.

No.	Rt	Q1	Q3	MW	Phenolic Compound	Concentration (µg/100 g dw)
1	1.28	168.9	125	170	Gallic Acid	152.32 ± 4.14
2	1.92	153	123	154	Hydroxytyrosol	22.06 ± 1.06
3	2.56	353	191	354	Chlorogenic Acid	32.26 ± 1.60
4	2.69	289	244.9	290	Catechin	62.42 ± 3.64
5	2.80	178.9	135	180	Caffeic Acid	152.32 ± 0.8
6	2.86	166.9	108	168	Vanillic Acid	148.5 ± 3.56
7	2.98	289	244.9	290	Epicatechin	101.56 ± 5.94
8	3.00	625.1	270.9	626	Quecetin-3,4-O-DG	89.90 ± 2.68
9	3.08	515.9	353	516	Cynarin	2.30 ± 0.14
10	3.29	609.2	299.9	610	Rutin	131.96 ± 2.98
11	3.35	163	119	164	4-Coumaric Acid	121.24 ± 2.36
12	3.46	463.1	300	464	Quercetin 3-O-G	34.68 ± 3.06
13	3.48	623.1	160.9	624	Verbascoside	3054.48 ± 31.16
14	3.52	593.2	284.9	594	Kaempferol 3-O-R	37.04 ± 2.12
15	3.54	389.1	227	390	Piceid	≤LOD
16	3.65	193	134	194	t-Ferulic acid	8.92 ± 0.50
17	3.69	447.1	284.1	448	Kaempferol 3-O-G	8.06 ± 1.30
18	3.85	447.1	284.9	448	Kaempferol 7-O-G	6.20 ± 1.48
19	3.97	359	161	360	Rosmarinic Acid	16.02 ± 1.18
20	4.01	435.1	272.9	436	Phloridzin	≤LOD
21	4.09	539.1	275	540	Oleuropein	269.26 ± 5.62
22	4.42	523.1	291	524	Ligstroside	43.44 ± 1.96
23	4.46	227.1	185	228	Resveratrol	≤LOD
24	4.60	284.9	133	286	Luteolin	10.40 ± 0.68
25	4.64	301	150.9	302	Quercetin	59.48 ± 1.86
26	5.02	268.9	117	270	Apigenin	1.62 ± 0.12
27	5.04	273	167	274	Phloretin	329.5 ± 6.80
28	5.13	270.9	150.9	272	Naringenin	2.42 ± 0.28

LOD, limit of detection. Values are reported as means ± s.d.

## Data Availability

Data are contained within the article.
